# Rapid T_1_ quantification from high resolution 3D data with model‐based reconstruction

**DOI:** 10.1002/mrm.27502

**Published:** 2018-10-22

**Authors:** Oliver Maier, Jasper Schoormans, Matthias Schloegl, Gustav J. Strijkers, Andreas Lesch, Thomas Benkert, Tobias Block, Bram F. Coolen, Kristian Bredies, Rudolf Stollberger

**Affiliations:** ^1^ Institute of Medical Engineering Graz University of Technology Graz Austria; ^2^ BioTechMed‐Graz Graz Austria; ^3^ Department of Biomedical Engineering and Physics Academic Medical Center Amsterdam Zuidoost The Netherlands; ^4^ Center for Advanced Imaging Innovation and Research New York University School of Medicine New York New York; ^5^ Bernard and Irene Schwartz Center for Biomedical Imaging New York University School of Medicine New York New York; ^6^ Institute for Mathematics and Scientific Computing University of Graz Graz Austria

**Keywords:** constrained reconstruction, inversion‐recovery Look‐Locker, imaging, model‐based reconstruction, MRI, T1 quantification, variable flip angle

## Abstract

**Purpose:**

Magnetic resonance imaging protocols for the assessment of quantitative information suffer from long acquisition times since multiple measurements in a parametric dimension are required. To facilitate the clinical applicability, accelerating the acquisition is of high importance. To this end, we propose a model‐based optimization framework in conjunction with undersampling 3D radial stack‐of‐stars data.

**Theory and Methods:**

High resolution 3D *T*_1_ maps are generated from subsampled data by employing model‐based reconstruction combined with a regularization functional, coupling information from the spatial and parametric dimension, to exploit redundancies in the acquired parameter encodings and across parameter maps. To cope with the resulting non‐linear, non‐differentiable optimization problem, we propose a solution strategy based on the iteratively regularized Gauss‐Newton method. The importance of 3D‐spectral regularization is demonstrated by a comparison to 2D‐spectral regularized results. The algorithm is validated for the variable flip angle (VFA) and inversion recovery Look‐Locker (IRLL) method on numerical simulated data, MRI phantoms, and in vivo data.

**Results:**

Evaluation of the proposed method using numerical simulations and phantom scans shows excellent quantitative agreement and image quality. *T*_1_ maps from accelerated 3D in vivo measurements, e.g. 1.8 s/slice with the VFA method, are in high accordance with fully sampled reference reconstructions.

**Conclusions:**

The proposed algorithm is able to recover *T*_1_ maps with an isotropic resolution of 1 mm^3^ from highly undersampled radial data by exploiting structural similarities in the imaging volume and across parameter maps.

## 1 INTRODUCTION

Quantitative MRI (qMRI) is a promising tool for precision medicine and offers the possibility to classify diseases based on physical quantities. Challenges for clinical applications include prolonged scan time and partial volume effects,^1^ in particular for focal lesions. Because in classical qMRI^2^, ^3^ several images of the same anatomical region are needed, the clinical applicability has been limited so far, especially when imaging large volumes. To overcome these limitations, acceleration of the data acquisition is essential. Since modern MRI scanners operate at the limits for peripheral nerve stimulation (PNS) and energy deposition (SAR), the most important strategy for further acceleration is reducing the number of encoding steps.

In recent years, many reconstruction techniques have been presented for recovering high quality images from incomplete data. Initially, these methods were based on the parallel imaging principle,^4^, ^5^ which synthesizes missing *k*‐space data from independent receiver coil information. In a next step, compressed sensing theory^6^, ^7^ had been integrated, which offers the possibility to incorporate a priori information on image sparsity in an iterative reconstruction process and can be synergistically combined with parallel imaging to further enhance the reconstruction quality with improved noise suppression. When used for qMRI, the quantitative maps are obtained from the recovered images with a pixel‐wise fitting approach.

However, in qMRI specific a priori information is usually available in the from of analytical signal models derived from the Bloch equations. The inclusion of explicit models into the reconstruction problem with the goal of directly estimating quantitative maps is commonly referred to as *model‐based reconstruction*. This method has been successfully applied to various parameter quantification problems,^8^, ^9^, ^10^, ^11^, ^12^, ^13^, ^14^, ^15^ demonstrating the potential for shortening the acquisition time while maintaining excellent quantification accuracy and high resolution. The model‐based reconstruction approaches aim to solve an inverse problem, inferring on the unknown NMR tissue parameters, using iterative optimization techniques. The problem typically consists of a non‐linear and often non‐convex data consistency term, composed of the forward operator and a pulse sequence specific signal equation. Classic model‐based schemes^8^, ^11^, ^14^, ^16^ employ smooth regularization functionals directly on the parameter maps rather than reconstructing the image series for each imaging parameter variation individually. In contrast to that, Tran‐Gia et al.^10^, ^12^ proposed to alternate between a pixel‐wise fit in image space and a data consistency step in the measured *k*‐space with no explicit regularization strategy. Doneva et al.^9^ proposed a different approach to model‐based reconstruction by utilizing the signal model to compute an overcomplete dictionary that is subsequently used as sparsifying transform in the reconstruction process. This can be seen as a first step towards the recently proposed iterative reconstruction for Magnetic Resonance Fingerprinting (MRF).^17^, ^18^, ^19^ Other approaches impose locally low rank constraints in parametric dimension,^20^ or a combination of low rank and sparsity constraints^21^, ^22^ on the image series and reconstruct the parameter maps in a second step from a non‐linear least squares problem. The advantage of such approaches is that the image reconstruction problem is convex and no assumptions on the involved relaxation process are necessary, e.g. mono‐ vs. multi‐exponential. The selection of the rank *L* can introduce small errors in the reconstructed maps^22^ but an additional reference data set can be used to determine *L* and translate it to data sets with similar acquisition parameters.^21^ Further, the estimation of the parameter maps still involves solving a non‐convex, non‐linear problem. The common optimization strategy for the model‐based reconstruction methods^11^, ^16^ is based on the non‐linear conjugate gradient (CG) algorithm.^23^, ^24^ This approach only allows for smooth regularization techniques, which insufficiently describe the structural information contained in the parameter maps. Recently, iteratively regularized Gauss‐Newton (IRGN) methods have been applied in the context of model‐based reconstruction problems.^14^, ^15^, ^25^ Combined with smooth regularization,^14^ the subproblem can be solved using a CG algorithm but IRGN can be combined with regularization terms enforcing more specific a priori knowledge on the parameter maps, e.g. joint sparsity using wavelets.^15^ Such regularization terms can be added to further improve the reconstruction quality but require different optimization approaches due to their non‐differentiability, e.g. fast iterative shrinkage‐thresholding algorithm (FISTA).^26^

In contrast to previous work,^11^, ^12^, ^14^, ^15^, ^16^, ^25^ we propose the following improvements. Firstly, more specific a priori information is introduced by means of the concept of Total‐Generalized‐Variation (TGV)‐based regularization,^27^ which was shown to yield improved noise suppression, especially for low SNR situations,^28^, ^29^ as compared to Total‐Variation. Furthermore, the TGV functional is adapted to the multiparametric setting by means of a Frobenius‐norm‐type TGV functional,^30^ which exploits structural information and shared features across parameter maps. Secondly, the optimization is applied to volumetric data in order to exploit structural information not only between parameter maps but also within the complete imaging volume. This allows for higher acceleration of the data acquisition while maintaining high accuracy of the parameter maps. The performance of the proposed reconstruction framework was analyzed for *T*_1_ quantification from 3D golden‐angle radial VIBE (RAVE)^31^ data, utilizing the variable flip angle (VFA) method,^3^ as well as for 3D golden‐angle radial stack‐of‐stars Inversion‐Recovery Look‐Locker (IRLL)^32^, ^33^ data. The agreement of undersampled VFA data to numerically simulated references was evaluated to investigate the gain in image quality between 2D and 3D regularization and show the advantages of joining the TGV functionals. Subsequently, MRI phantom measurements for VFA and IRLL data, acquired in a prospective manner, were compared to fully‐sampled reference reconstructions, by fitting the non‐linear model in image space. Finally, accelerated in vivo brain measurements from healthy volunteers were compared to established references for both models, studying the gain in image quality between 2D and 3D regularization.

## 2 THEORY

We denote by *N*_*x*_, *N*_*y*_ and *N*_*z*_ the dimensions of image space $U\;=\;C^{N_{x}\;\;\times \;\;N_{y}\;\;\times \;\;N_{z}}$, by $N_{p}$ the number of imaging parameter encodings, and by $N_{c}$ the number of coils. Denoted by *N*_*d*_ is the number of *k*‐space encodings defining the data space as $V\;=\;C^{N_{p}\;\;\times \;\;N_{c}\;\;\times \;\;N_{d}}$. Further we define $N_{u}$ as the number of unknown tissue parameters and *N*_∇_ as the number of spatial derivative directions, i.e. the *x*,* y* and *z* direction.

### 2.1 Iteratively regularized Gauss‐Newton with Frobenius total generalized variation constraint

Model‐based parameter quantification utilizes an analytical relationship of the MR signal $S_{p}\left(u\right)$ to the unknown tissue parameter maps of interest, e.g. $u\;=\;\left(M_{0},T_{1}\right)\;\in \;U^{2}$, to quantify the latter. $S_{p}\left(u\right)$ varies depending on the used imaging sequence with the imaging parameters *p* and is included in the MR signal equation to map to the acquired multicoil, multiparametric *k*‐space data *d*_*p*,*c*_. The basic MR signal equation modulates the magnetization $m_{p}\;=\;S_{p}\left(u\right)$ for the p‐th acquisition with the receiver coil sensitivities *b*_*c*_ for receive‐channel *c*, followed by (non‐uniform) Fourier encoding $F_{p}$. Here, index *p* denotes a variable *k*‐space coverage for each parameter acquisition, such that the final non‐linear forward mapping A can be written as follows:

(1) $$A:\;u\mapsto {\left(F_{p}\left\{b_{c}S_{p}\left(u\right)\right\}\right)}_{p,c}.$$

The resulting cost function to identify the unknown parameters *u* is given by

(2) $$\min_{u}\frac{1}{2}{\left\|A\left(u\right)-d\right\|}_{2}^{2}+\lambda R\left(u\right)$$

and comprises a $L^{2}$‐data fidelity term, which measures the mismatch between the forward model in Equation (1) and acquired data. Use of the *L*_2_ norm is justified by the well‐known normally distributed noise statistics in *k*‐space data. The typically bi‐linear forward mapping A leads to a non‐convex data fidelity term. Due to additional undersampling, this reconstruction problem is ill‐posed and, hence, the regularization term R plays a crucial role. A proper value needs to be chosen for the parameter λ to balance between data fidelity term and regularization. To exploit additional information, advanced regularization terms need to be introduced that reflect specific a priori knowledge. Typical assumptions about natural and medical images are that the images are composed of regions with smooth or linear varying contrast, separated by sharp edges. Therefore, we replace R(u) by a well‐studied functional reflecting these properties, namely the 2^nd^‐order Total Generalized Variation (TGV^2^).^27^ It has been shown that TGV^2^ yields excellent image quality for reconstructed images from undersampled MR data.^28^ To additionally exploit shared features between the parameters of interest, the TGV^2^ functionals are joined using a Frobenius norm, which will be used throughout the paper and denoted as R(u). The TGV${}_{\text{Frob}}^{2}$ regularization itself is characterized as minimization problem of the following form

(3) $${\text{TGV}}_{\text{Frob}}^{2}\left(u\right)\;=\;\min_{v}\;\alpha_{1}{\left\|\nabla u-v\right\|}_{1,2,2}+\alpha_{0}{\left\|Ev\right\|}_{1,2,2},$$

where α_0_ and α_1_ are chosen to balance between the first and second derivative information, ∇ corresponds to finite forward differences and E denotes the symmetrized derivative $Ev\;=\;\frac{1}{2}\left(\nabla v\;+\;\nabla v^{T}\right)$. Here, we abuse the notation of the norm ‖·‖_1,2,2_ defined for $v\;=\;{\left(v^{1,i},v^{2,i},v^{3,i}\right)}_{i\;=\;1}^{N_{u}}\;\in \;U^{3\;\;\times \;\;N_{u}}$ as

$${\left\|v\right\|}_{1,2,2}\;=\;\sum_{j,k,l}^{N_{x},N_{y},N_{z}}\sqrt{\sum_{i}^{N_{u}}|v_{j,k,l}^{1,i}{|}^{2}+|v_{j,k,l}^{2,i}{|}^{2}+{\left|v_{j,k,l}^{3,i}\right|}^{2}}$$

and for $\xi \;=\;{\left(\xi^{1,\;i},\;\xi^{2,\;i},\;\xi^{3,\;i},\;\xi^{4,\;i},\;\xi^{5,\;i},\;\xi^{6,\;i}\right)}_{i\;=\;1}^{N_{u}}\;\in \;U^{6\;\times \;N_{u}}$ as

$${\left\|\xi \right\|}_{1,\;2,\;2}\;=\;\sum_{j,\;k,\;l}^{N_{x},\;N_{y},\;N_{z}}\sqrt{\sum_{i}^{N_{u}}|\xi_{j,\;k,\;l}^{1,\;i}{|}^{2}+|\xi_{j,\;k,\;l}^{2,\;i}{|}^{2}+|\xi_{j,\;k,\;l}^{3,\;i}{|}^{2}+2|\xi_{j,k,l}^{4,i}{|}^{2}+2|\xi_{j,\;k,\;l}^{5,i}{|}^{2}+2{\left|\xi_{j,\;k,\;l}^{6,\;i}\right|}^{2}},$$

where the factor 2 in front of *ξ* ^4, *i*^, *ξ* ^5, *i*^, *ξ* ^6, *i*^ compensates for the symmetrization of the Jacobian in the definition of E. Incorporating this functional in Equation (2) yields the following optimization problem:

(4) $$\min_{u,v}\;\frac{1}{2}{\left\|A\left(u\right)-d\right\|}_{2}^{2}+\lambda \left(\alpha_{1}{\left\|\nabla u-v\right\|}_{1,\;2,\;2}+\alpha_{0}{\left\|Ev\right\|}_{1,\;2,\;2}\right).$$

### 2.2 Numerical solution

The optimization problem in Equation (4) is difficult to solve for several reasons. The non‐differentiability prevents the use of first‐order derivative‐based optimization algorithms, e.g. non‐linear CG, and the non‐linear operator A further limits the applicable solution strategies. Therefore, a solution strategy based on the Gauss‐Newton approach is applied, i.e linearizing A with respect to *u* around *u*_*k*_ as

(5) $$A\left(u\right)\approx A\left(u_{k}\right)+\frac{\partial A}{\partial u}{|}_{u\;=\;u_{k}}\left(u-u_{k}\right),$$

such that Equation (4) is solved by iterating sufficient Gauss‐Newton steps with convex inner subproblems of the form

(6) $${\hat{u}}_{k}\;=\;\underset{u,v}{\arg\min}\;\;\frac{1}{2}\|\text{DA}u-d_{k}{\|}_{2}^{2}+\lambda \left(\alpha_{1}{\left\|\nabla u-v\right\|}_{1,2,2})+\alpha_{0}{\left\|Ev\right\|}_{1,2,2}\right)+\frac{1}{2\gamma }{\left\|u-u_{k}\right\|}_{2}^{2}.$$

It was shown by Salzo et al.^34^ that the GN approach converges with linear rate to a critical point for non‐convex problems with non‐differentiable penalty functions if initialization is sufficiently close. Here, the additional *L*_2_ norm on (*u*−*u*_*k*_) serves as step size penalty and improves the convexity of the subproblem. Constant terms, stemming from the linearization, are combined with the *k*‐space data, i.e. $d_{k}\;=\;d-A\left(u_{k}\right)\;+\;\text{DA}u_{k}$ and the matrix $\text{DA}\;=\;\frac{\partial A}{\partial u}{|}_{u\;=\;u_{k}}\left(u\right)$ is precomputed at each linearization step. Equation (6) can be related to a saddle point problem of the form

(7) $$\min_{x}\;\max_{y}〈\text{K}x,y〉+G\left(x\right)-F^{*}\left(y\right),$$

that can be efficiently solved with the primal‐dual (PD) algorithm described in.^35^ Here, *K* is a linear operator, *F* and *G* are convex, lower semi‐continuous functionals, and F* denotes the convex conjugate of *F*. The required saddle point formulation in Equation (7) for Equation (6) can be obtained using the convex conjugate as follows:

$$\begin{array}{cc} & \Leftrightarrow \min_{x\;=\;(u,v)}\frac{1}{2}\|\text{DA}u-d_{k}{\|}_{2}^{2}+\lambda \left(\alpha_{1}{\left\|\nabla u-v\right\|}_{1,2,2}+\alpha_{0}{\left\|Ev\right\|}_{1,2,2}\right)+\frac{1}{2\gamma }{\left\|u-u_{k}\right\|}_{2}^{2}\\ & \;=\;\min_{x\;=\;(u,v)}\max_{y\;=\;(z,r)}〈\text{DA}u,r〉-〈d_{k},r〉-\frac{1}{2}{\left\|r\right\|}_{2}^{2}+\left(K_{1}x,z\right)-I_{\left\{\|\cdot {\|}_{\infty \leq \alpha_{0}\lambda ,\alpha_{1}\lambda }\right\}}\left(z\right)+\frac{1}{2\gamma }{\left\|u-u_{k}\right\|}_{2}^{2}\\ & \;=\;\min_{x\;=\;(u,v)}\max_{y\;=\;(z,r)}\;〈\text{K}x,y〉+G\left(x\right)-F*\left(y\right) \end{array}.$$

with $K\;=\;\left(\begin{array}{cc} \text{DA} & 0\\ & K_{1} \end{array}\right),$ $K_{1}\;=\;\left(\begin{array}{cc} \nabla & -id\\ 0 & E \end{array}\right),\;F*\left(y\right)\;=\;F*\left(z,r\right)\;=\;〈d_{k},r〉\;+\;\frac{1}{2}{\left\|r\right\|}_{2}^{2}+I_{\left\{\|\cdot {\|}_{\infty \leq \alpha_{0}\lambda ,\alpha_{1}\lambda }\right\}}\left(z\right)$, and $G\left(x\right)\;=\;G\left(u\right)\;=\;\frac{1}{2\gamma }{\left\|u-u_{k}\right\|}_{2}^{2}.$ DA is the Jacobian matrix at *u*_*k*_ of the non‐linear MR signal equation for all scans *p*, unknowns *i* and coils *c*:

(8) $$\text{DA}:u\;=\;{\left(u_{i}\right)}_{i\;=\;1}^{N_{u}}\to {\left(\sum_{i}F_{p}\left[b_{c}{\left.\frac{\partial S_{p}\left(u\right)}{\partial u_{i}}\right|}_{u\;=\;u_{k}}u_{i}\right]\right)}_{p\;=\;1}^{N_{p}}\;=\;{\left(\xi_{p}\right)}_{p\;=\;1}^{N_{p}}.$$

The update scheme for the PD algorithm is given in Algorithm 1 as pseudo code.

To speed up the convergence of the PD algorithm, a recently proposed implementation with line search is used.^36^ Computational complexity of one GN step of the algorithm amounts to $O(N_{p}N_{c}N_{z}\left(N_{d}\log N_{d}\right)\;+\;N_{u}N_{z}N_{x}N_{y})$, the complete complexity analysis is shown in the supporting material as Supporting Information Text S1. The algorithm is terminated if either a predetermined number of steps is reached, a convergence criterion is fulfilled, or stagnation is detected. For convergence, the energy decrease in the primal problem as well as in the primal‐dual gap are monitored and the algorithm terminates if changes in the energy are below a predetermined threshold. The regularization parameters λ and γ are altered after each inner iteration to reduce regularization over the course of minimization, leading to stronger regularization at the initial iterations and more data weighting at the end. Changing the regularization parameters during optimization can have serious impact on convergence and speed of the algorithm and has been shown to be beneficial in the context of IRGN methods.^37^ For comparison, we also implemented a TV and *L*_1_‐wavelet, constrained imposed on each parameter map, by replacing H in Equation (8) with a gradient respectively Daubechies‐4 wavelet transformation from PyWavelets,^38^ the same wavelet type as used by Wang et al.^15^

(9) $${\hat{u}}_{k}\;=\;\underset{u}{\arg\min}\;\;\frac{1}{2}\|\text{DA}u-d_{k}{\|}_{2}^{2}+{\lambda \|\text{H}u\|}_{1,2,2}+\frac{1}{2\gamma }{\left\|u-u_{k}\right\|}_{2}^{2}.$$

For optimization, the same PD algorithm (Algorithm 2) as in the TGV case is used. For the *L* ^1^‐wavelet regularization, the norm ‖·‖_1, 2, 2_ is computed for all levels. The number of levels was determined in 2D by the PyWavelets toolbox.

Additionally a seperate regularization approach for all regularization strategies is employed by changing the definition of the norm ‖·‖_1, 2, 2_ to ‖·‖_1, 2_ given by

$${\left\|v\right\|}_{1,\;2}\;=\;\sum_{i}^{N_{u}}\sum_{j,\;k,\;l}^{N_{x},\;N_{y},\;N_{z}}\sqrt{|v_{j,\;k,\;l}^{1,\;i}{|}^{2}+|v_{j,\;k,\;l}^{2,\;i}{|}^{2}+{\left|v_{j,\;k,\;l}^{3,\;i}\right|}^{2}}.$$

### 2.3 The application to T1 quantification

In the present work, two commonly used models are implemented to quantify *T*_1_ from radially acquired 3D data, the VFA‐*T*_1_ approach^3^, ^39^ as well as the model for the IRLL sequence.^32^, ^40^

The VFA‐*T*_1_ model describes the signal intensity in dependency on the flip angle α and *T*_*R*_ for a RF and gradient spoiled gradient echo sequence (FLASH) and is given by

(10) $$S_{\alpha }^{\mathrm{VFA}}\left(M_{0},T_{1}\right)\;=\;M_{0}\sin\alpha \frac{1-E_{1}}{1-E_{1}\cos\alpha }$$

with

$$E_{1}\;=\;e^{\frac{-T_{R}}{T_{1}}}.$$

The measurements were carried out with fixed *T*_*R*_ for a defined range of flip angle variations (α_*p*_). Literature suggestions on optimal flip angle selection for the VFA technique vary. We used a set of 10 flip angles ranging from 1^∘^ to 19^∘^ in 2^∘^ steps, as suggested in.^41^ *T*_*E*_ was kept as short as possible to reduce T_2_* effects, which were subsequently neglected. The method is known to be sensitive to transmit field (B1^+^) inhomogeneities. Therefore, additional flip angle mapping was performed.

The second investigated *T*_1_ mapping procedure, the IRLL technique, is based on an inversion pulse followed by a train of *N* read‐out pulses with a fixed small flip angle α. The model depends on the time delay *t*_*d*_ between the inversion pulse and the first α pulse, the time between subsequent pulses *τ* and an optional time *t*_*r*_ at the end of the pulse train, describing a recovery of longitudinal relaxation to equilibrium. The signal intensity *S*_*n*_ of the *n*th gradient echo read out can be described as^32^, ^33^, ^40^

(11) $$S_{n}^{\text{IRLL}}\left(M_{0},T_{1}\right)\;=\;\sin\alpha M_{0}\left[F+{\left(\cos\alpha \right)}^{n-1}\left(Q-F\right)\right]$$

with

$$\begin{array}{cc} F & \;=\;\frac{1-E_{\tau }}{1-\cos\alpha E_{\tau }}\\ Q & \;=\;\frac{-F\cos\alpha E_{r}E_{d}\left[1-{\left(\cos\alpha E_{\tau }\right)}^{N-1}\right]-2E_{d}+E_{r}+1}{1+\cos\alpha E_{r}E_{d}{\left(\cos\alpha E_{\tau }\right)}^{N-1}}\\ E_{\tau } & \;=\;\exp\left(\frac{-\tau }{T_{1}}\right);\;\;\;\;E_{r}\;=\;\exp\left(\frac{-t_{r}}{T_{1}}\right);\;\;\;\;E_{d}\;=\;\exp\left(\frac{-t_{d}}{T_{1}}\right) \end{array}.$$

Here, *N* is the total number of acquired readouts. In general, α, *τ*, and *t*_*d*_ should be kept as short as possible.^33^ Furthermore, the signal equation assumes a perfect inversion. However, models exists that take non perfect inversion into account.^32^ In the special case of radial acquired single shot IRLL data, the model equation is valid for every acquired spoke. This can be used to achieve a certain amount of temporal resolution by binning a predefined number of spokes into one *k*‐space. The forward computation consists of evaluating the model for every acquired spoke, followed by mean value calculation over the computed images according to the number of combined spokes per *k*‐space. For in‐vivo data 13 spokes are combined together, which has been shown to be a suitable trade‐off between temporal fidelity and computational burden,^14^ for phantom data 5 spokes are combined to enhance temporal resolution.

## 3 METHODS

### 3.1 Data preprocessing

To improve the general applicability of the algorithm, the acquired *k*‐space data was normalized by its $L^{2}$‐norm. This leads to an almost measurement independent data norm and allows for use of uniform regularization parameters across different measurements. Scaling was chosen to yield a data $L^{2}$‐norm of 1000 times the square root of slices. Balancing the partial derivatives was achieved by introducing scale factors for each unknown separately. Proton density scale was fixed to match the mean of the acquired signal curve for a simulated range of *T*_1_ from 10 ms to 5000 ms. *T*_1_ scale was adjusted after each GN step keep the partial derivatives balanced. Additionally, the numerical gradients were balanced after each GN‐step by calculating their $L^{2}$‐norm and applying a scaling to match each other in the forward and adjoint evaluation of the gradient. In a subsequent preprocessing step, coil sensitivities were estimated from parameter‐averaged data using the method of Uecker et al.^42^ Deviations from the nominal flip angle were taken into account by performing $B_{1}^{+}$ mapping with a modified Bloch‐Siegert^43^, ^44^ and DREAM^45^ method for VFA respectively IRLL data. The Bloch‐Siegert map was acquired on a Cartesian grid with half the resolution of the VFA data and a block undersampling pattern with size of 12 × 4 as described by Lesch et al.,^43^ leading to an acquisition time of about 15s for the whole 3D volume. The resulting $B_{1}^{+}$ map is normalized to the nominal flip angle of the Bloch‐Siegert encoding pulse, leading to a spatially dependent correction factor for α. The DREAM mapping was acquired similarly and employed the following parameters: STEAM angle 60^∘^, *T*_*R*_/*T*_*E*1_/*T*_*E*2_  =  4.5/1.5/2.1 ms, acquisition time: 4 s, resolution 3.2 × 3.2 × 4 mm^3^. Subsequently correction maps were interpolated to match the resolution of the acquired measurements.

### 3.2 Simulation studies

Numerical *T*_1_ and (pseudo) proton density *M*_0_ brain phantoms were used as ground truth for the simulation studies, which are part of the MRiLab toolbox^46^ for MATLAB (The Mathworks, Natick, Massachusetts). *T*_1_ and *M*_0_ values were chosen to agree with in vivo brain structures for 3T. Additional, a tumor was simulated as an 3D elliptical structure with linearly varying contrast in white matter. By applying the signal equations introduced in the Theory section, image data was generated with the corresponding VFA forward model in Equation [Link] and modulated with seven artificial coil sensitivity profiles, generated using Biot‐Savart's law. The synthetic coil images were Fourier‐transformed and resampled along a specified Fibonacci‐number of radial spokes according to the golden‐angle (111.25^∘^) scheme.^47^ The golden‐angle scheme was continued over all simulated scans, see Supporting Information Figure S1. Matrix sizes for the VFA phantoms were set to 216 × 216 voxels and transformed using a non‐uniform FFT to yield 432 read‐out samples per spoke and 34 spokes per flip angle variation, simulating undersampled acquisition. The number of spokes was chosen according to the Fibonacci series to achieve uniform *k*‐space coverage. Simulated flip angles ranged from 1^∘^ to 19^∘^ in 2^∘^ steps, resulting in 10 independent simulations. A *T*_*R*_ of 5.38 ms was used throughout all simulations. Gaussian noise was added to the *k*‐space data, to emulate typical SNR levels for in vivo measurements.

All methods were terminated according to the described stopping criteria. Thirteen Gauss‐Newton iterations were run to ensure convergence to an optimal solution. The initial number of PD iterations was set to 100 and increased to 200 and 300 in the first GN steps, which was found to be sufficient for convergence.

### 3.3 MRI measurements of phantoms and healthy human volunteers

VFA measurements were performed on a clinical 3T MAGNETOM Skyra scanner (Siemens Healthineers, Erlangen, Germany), employing a 20‐channel head coil for phantom measurements and a 32‐channel head coil for in vivo acquisitions. The MRI phantom consists of 5 tubes filled with doped water, surrounded by tap water. VFA measurements were performed using the RAVE sequence with golden‐angle ordering scheme. The measurement protocol consisted of a fully‐sampled reference scan using 10 flip angles for standard reconstruction and fitting in image space, followed by accelerated scans. In the case of undersampled acquisition, the trajectory is rotated in the same fashion as in the simulation studies. Fully sampled acquisition consisted of 400 acquired spokes. Forty reference lines were acquired prior to all acquisitions to account for gradient system imperfections.^48^ For the MRI phantom measurements, a flip angle set ranging from 1^∘^ to 19^∘^ in 2^∘^ steps was acquired with a matrix size of 256 × 256 × 40 and 1 mm^3^ resolution followed by accelerated scans with 21, 13, and 8 spokes. Bandwidth was set to 490 Hz/Pixel and 25% oversampling in *k*_*z*_ direction with *T*_*R*_ = 5 ms and *T*_*E*_ = 2 ms. In vivo experiments were performed using the same flip angle range, matrix size, and 89 to 8 spokes. *T*_*R*_ was set to 5.5 ms and *T*_*E*_ to 2.46 ms to measure the first in‐phase echo. Furthermore, bandwidth was reduced to 340 Hz/Pixel to increase SNR.

The IRLL data was acquired on a 3T Philips Ingenia system (Philips Medical Systems, Eindhoven, The Netherlands) using a 16‐channel head coil. Measurements of the ISMRM‐NIST phantom^49^ and in vivo head data were acquired. The IRLL sequence consisted of an inversion pulse followed by a single‐shot readout using a radial stack‐of‐star FLASH sequence, which was repeated for every acquired *k*_*z*_ encoding. The delay between inversion and the first readout pulse was measured as 14.3 ms followed by a train of 5^∘^ readout pulses spaced 5.5 ms apart. A total number of 731 spokes were acquired after each inversion with a 212 × 212 × 40 scan matrix, 1 mm^3^ isotropic resolution, and 50% oversampling along *k*_*z*_. The total shot time per slice was chosen to be 8 seconds. For reconstruction, every 13 consecutive spokes were binned to reduce the overall computational burden while maintaining high temporal resolution. For validation and comparison purposes, a fully sampled 2D Cartesian reference measurement of the central slice was performed in 2 min and 41 s. The same protocol was used for phantom and in vivo measurements.

The reference reconstructions for the VFA and IRLL methods were generated with a model‐based fit in image space from fully‐sampled non‐Cartesian respectively Cartesian data with simple regridding and Fourier transformation or Fourier transformation. Different ROIs are specified to reflect white matter (WM), central and cortical gray matter (GM), and cerebrospinal fluid (CSF). For all in vivo applications, written informed consent was obtained from healthy volunteers in compliance with the guidelines from the local ethical commission.

### 3.4 Implementation

Reconstruction was performed offline using Python (Python Software Foundation, https://www.python.org/) and a gpuNUFFT^50^ wrapper from the Primal‐Dual toolbox^51^ on desktop PCs equipped with a Tesla K40 and GeForce GTX 1070 (NVIDIA, Santa Clara, California). The regularization parameters for the described algorithm were selected according to parameter training and are altered after every GN iteration. Reconstruction for all methods was started with λ = 10^−2^ and γ = 10^1^. The weight λ was decreased by a factor of *q*_λ_ = 0.7 and γ was increased by a factor of *q*_γ_ = 2 in order to avoid over‐regularization throughout the optimization. λ_min_ = 1.8 × 10^−3^/2.3 × 10^−3^/1 × 10^−2^ and γ_max_ = 10^2^ had been chosen as limits for numerical phantom data for TGV/TV/*L* ^1^‐wavelet regularization, λ_min_ = 1.8 × 10^−3^/2.3 × 10^−3^/3.7 × 10^−3^ for VFA Phantom and λ_min_ = 3.4 × 10^−3^/3.4 × 10^−3^/2.3 × 10^−3^ for VFA in‐vivo experiments. IRLL reconstructions were performed with λ_min_ = 3.7 × 10^−3^/3.7 × 10^−3^/2.3 × 10^−3^, γ_max_ = 10^2^ for MRI phantom and in vivo data. The model parameters for the TGV${}_{\text{Frob}}^{2}$ functional were set to α_1_/α_0_ = 1/2 equally for VFA and IRLL, which proved to be a reasonable choice for gray scale images.^28^ PD line‐search parameters were set to β = 400, the initial weight between primal and dual step size, and *μ* = 0.5, the reduction factor of the primal step size *τ*. The choice was taken from^36^“Algorithm 2” to assure compliance with ${\sigma \tau \|K\|}_{2}^{2}<1$, the necessary condition to guarantee convergence of the PD algorithm. The reconstruction was initialized with constant *M*_0_ = 1 and *T*_1_ = 800 ms. The influence of different *T*_1_ initialization was evaluated by randomly selecting a $T_{1}\;\in \;\left[200,\;5000\right]$ ms and running the algorithm 100 times, see Supporting Information Figure S2. The source code together with some examples can be found at: https://github.com/IMTtugraz

## 4 RESULTS

**Figure 1:**
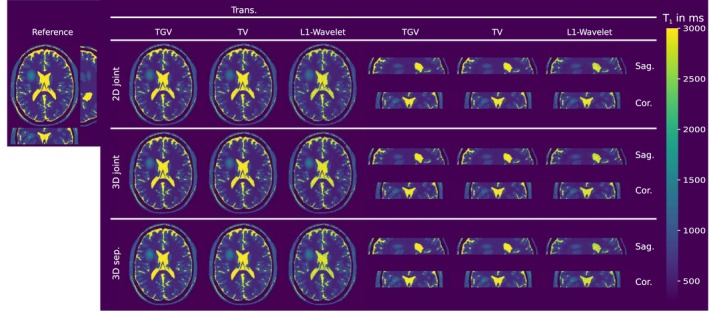
Numerical simulated VFA *T*_1_ reconstructions with 34 simulated spokes compared to a numerical reference in the top left. *T*_1_ values are given in milliseconds. Shown are transversal, sagittal and coronal views of the phantom with a simulated “tumor” in the white matter. The corresponding relative absolute error is given in Figure 2

**Figure 2:**
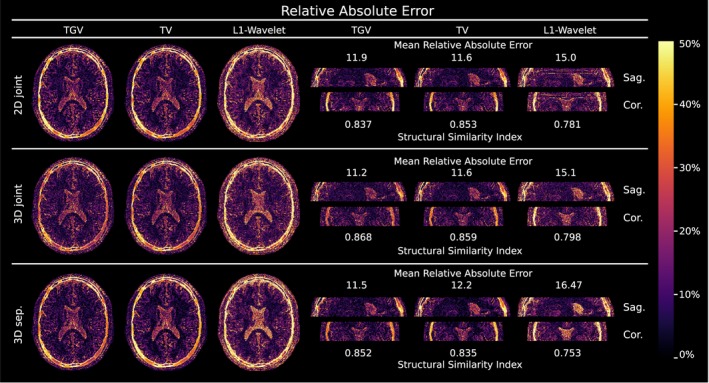
Relative absolute error of numerical simulated VFA *T*_1_ reconstructions with 34 simulated spokes compared to a numerical reference in.^1^ Shown are transversal, sagittal and coronal views of the phantom with a simulated “tumor” in the white matter. The error is given given in percent. The numbers next to the images indicate the mean relative absolute error in the corresponding parameter maps as well as the structural similarity index

**Figure 3:**
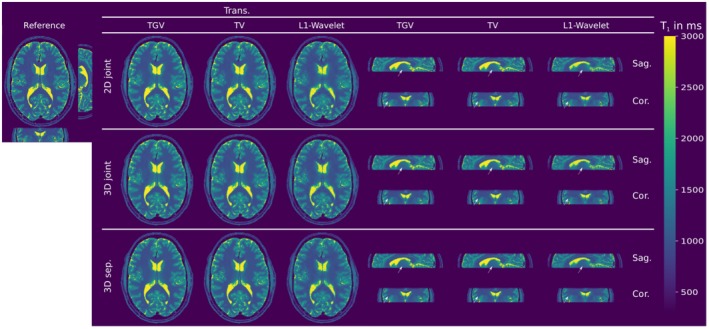
Comparison of 3D to 2D regularization for in vivo VFA *T*_1_ reconstruction from 34 acquired spokes. *T*_1_ maps are given in milliseconds. Visually observable differences are marked with white arrows. The corresponding relative absolute error maps are given in Supporting Information Figure S3. The skull has been masked out

**Figure 4:**
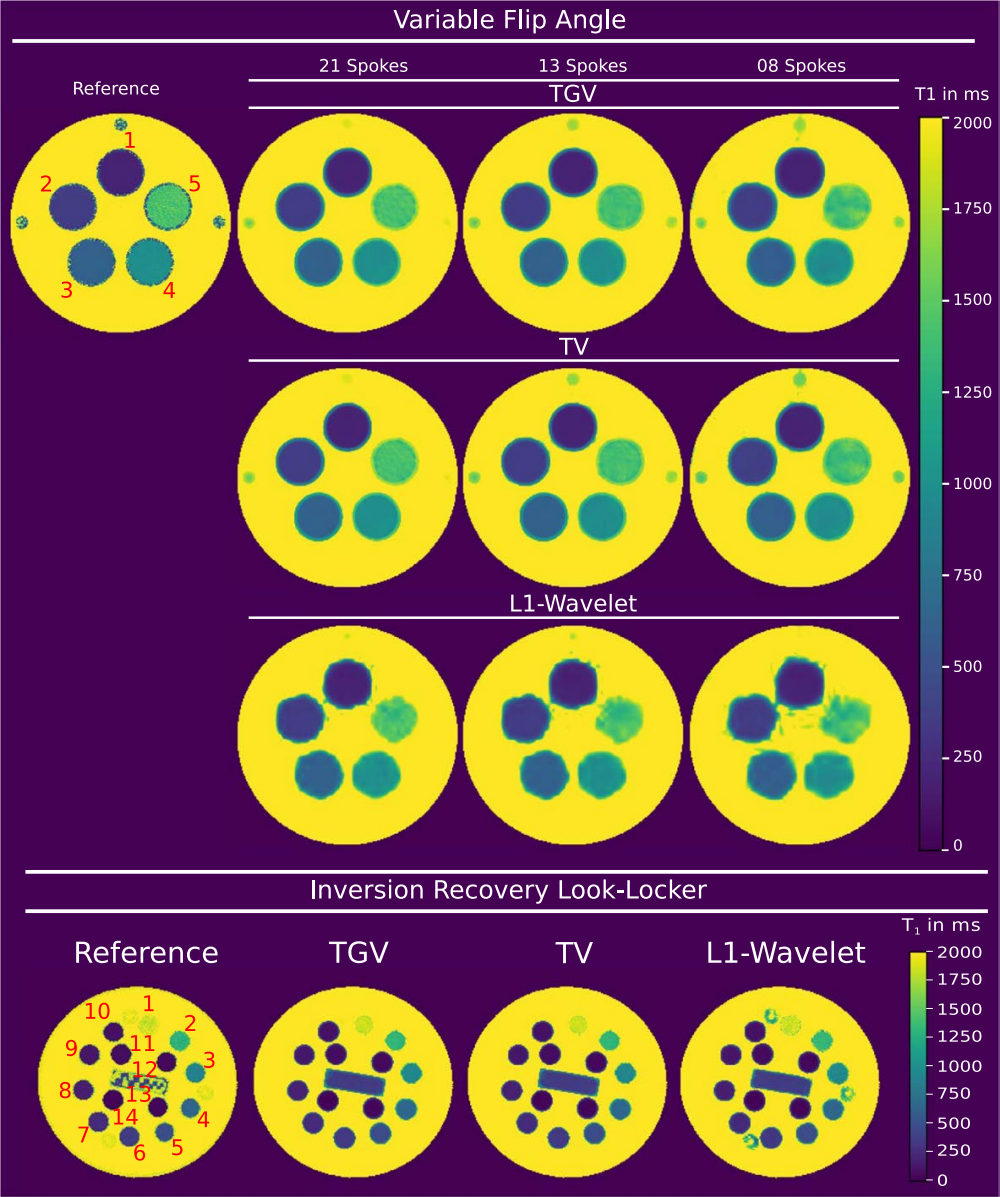
MRI phantom *T*_1_ measurements using a VFA based sequence are shown in the upper part. Fully sampled reference in the top left, compared to the proposed method in the 1st row, TV regularization in the 2nd row, and *L* ^1^‐wavelet regularization in the 3rd row. The lower part shows MRI phantom reconstructions using a IRLL based sequence. Cartesian reference in the lower left, compared to the proposed method and TV respectively *L* ^1^‐wavelet regularization. All values are given in milliseconds. Quantitative evaluation of ROIs, marked in the reference, are given in Table 1 for VFA and Table 2 for IRLL reconstructions

Figure 1 shows the comparison of simulated MRI phantom reconstructions from undersampled VFA data to the numerical *T*_1_ reference. Visually, no difference is observable between 2D and 3D, respectively joint and separate regularization. TV and TGV regularized results show lower noise levels than *L* ^1^‐wavelet results. The difference between the regularization strategies becomes evident by looking at corresponding error maps in Figure 2 and the mean relative absolute error (MRAE) respecively structural similarity index (SSIM) of the whole volume. Joint 3D TGV regularization performs best with a MRAE of 11.2% and SSIM of 0.868 compared to the 2nd best result of joint 3D TV with a MRAE of 11.6% and SSIM of 0.859. For tissue with little to no signal, e.g. the skull, larger differences can be observed. Reconstructions from 34 spokes of VFA in vivo data in Figure 3 show the influence of 3D versus 2D regularization and joint versus separate regularization. In the reformatted sagittal views, artifacts in slice direction are visible in the 2D reconstruction, marked with white arrows. Error maps in Supporting Information Figure S3 show a good agreement of all methods except for CSF boundaries and vessels to the reference reconstruction. 3D reconstruction decreases the MRAE and improves the SSIM in cases of TV and TGV based regularization, however, SSIM decreases for 3D reconstruction using wavelets, as shown in Supporting Information Figure S3. Again, TGV and TV reconstructions lead to improved noise suppression as compared to wavelet based regularization. The reconstruction results obtained from accelerated VFA MRI phantom measurements with 3D TGV, TV, and wavelet regularization are compared in Figure 4 to a fully sampled reference. Mean *T*_1_ values with the corresponding standard deviation (SD) were computed from marked and numbered ROIs for all reconstructions and are summarized in Table 1. SD of TGV and TV based results are lower compared to wavelet based results which also agrees with the visual impression in Figure 4. Computed mean *T*_1_ values from all reconstructions exhibit good compliance with the reference, lying within one to two SD of mean *T*_1_ values of the reference. However, the reconstruction from only eight spokes exhibits increased blurring in areas with little to no signal intensity, e.g. at the boundary of the tubes. *L* ^1^‐wavelet reconstructions are corrupted with artefacts at the border of the tubes for all cases compared to TV and TGV based results. Similar results are obtained from IRLL data from the ISMRM‐NIST phantom, as displayed in Figure 4. *T*_1_ maps are in agreement with the reference which is further supported by a comparison of quantitative values for 14 selected ROIs, provided in Table 2. Again, TGV and TV perform similar and both outperform wavelets regarding noise suppression.

**Table 1:** MRI phantom measurement results using the VFA method. Quantitative evaluation of specified ROIs in Figure 4. All values are given in ms as mean ± SD

	ROI 1	ROI 2	ROI 3	ROI 4	ROI 5
	Reference				
	199 ± 5	368 ± 10	634 ± 19	1012 ± 43	1437 ± 80
3D TGV^2^‐Frobenius					
21 Spokes	195 ± 1	362 ± 2	621 ± 7	998 ± 17	1433 ± 38
13 Spokes	200 ± 2	361 ± 2	622 ± 7	981 ± 20	1412 ± 37
08 Spokes	202 ± 1	373 ± 8	607 ± 12	978 ± 33	1415 ± 74
3D TV					
21 Spokes	202 ± 1	367 ± 2	624 ± 6	997 ± 15	1429 ± 33
13 Spokes	201 ± 1	361 ± 2	621 ± 7	985 ± 18	1412 ± 36
08 Spokes	204 ± 1	374 ± 5	606 ± 10	976 ± 27	1416 ± 64
*L* ^1^‐wavelet					
21 Spokes	195 ± 11	360 ± 14	622 ± 20	1010 ± 36	1425 ± 66
13 Spokes	193 ± 13	353 ± 15	619 ± 21	994 ± 33	1396 ± 78
08 Spokes	196 ± 12	362 ± 13	603 ± 24	992 ± 50	1391 ± 95

**Table 2:** MRI NIST phantom measurement results obtained with an IRLL sequence. Quantitative evaluation of the ROIs specified in Figure 4. All values are given in ms and as mean ± SD

ROI	1	2	3	4	5	6	7
Reference^49^	1838	1398	998	726	509	367	259
2D Cartesian	1842 ± 100	1283 ± 58	884 ± 45	650 ± 34	474 ± 30	356 ± 33	255 ± 21
8s 3D TGV${}_{\text{Frob}}^{2}$	1856 ± 56	1338 ± 33	944 ± 16	672 ± 7	477 ± 4	336 ± 4	239 ± 1
8s 3D TV	1857 ± 59	1336 ± 31	944 ± 16	672 ± 8	478 ± 4	337 ± 3	240 ± 1
8s *L* ^1^‐wavelet	1850 ± 110	1347 ± 69	959 ± 45	666 ± 27	477 ± 18	330 ± 16	236 ± 9
ROI	**8**	**9**	**10**	**11**	**12**	**13**	**14**
Reference^49^	185	131	91	64	46	33	23
2D Cartesian	176 ± 15	126 ± 9	93 ± 9	70 ± 9	49 ± 8	38 ± 7	26 ± 6
8s 3D TGV${}_{\text{Frob}}^{2}$	182 ± 2	130 ± 5	96 ± 2	68 ± 1	51 ± 2	38 ± 1	22 ± 4
8s 3D TV	183 ± 1	130 ± 4	96 ± 2	68 (0)	50 ± 1	38(0)	23 ± 2
8s *L* ^1^‐wavelet	174 ± 8	125 ± 10	95 ± 6	61 ± 4	46 ± 8	34 ± 7	21 ± 8

**Figure 5:**
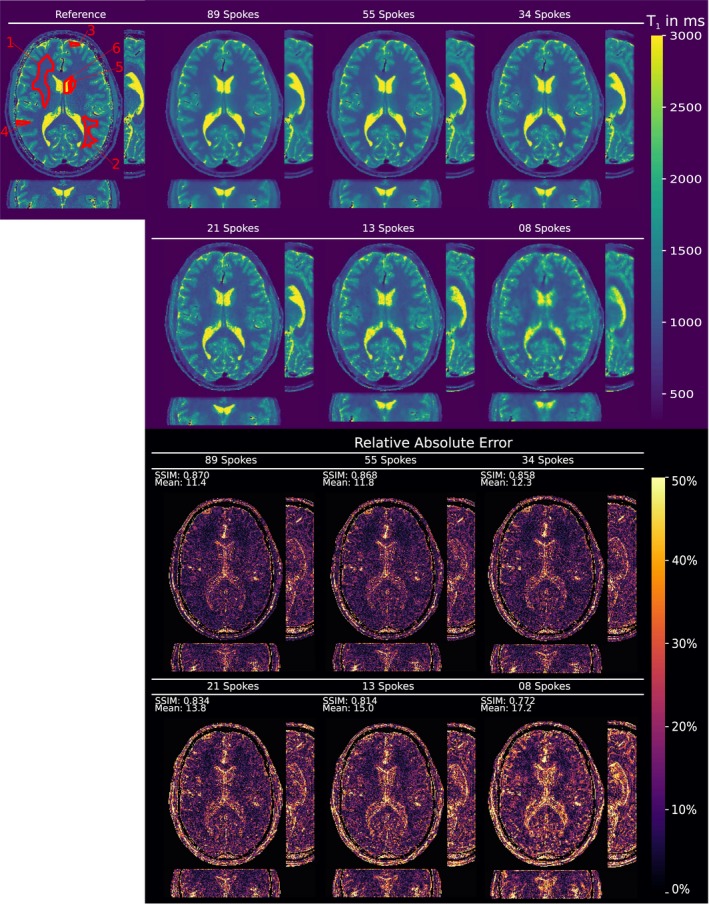
In vivo VFA *T*_1_ measurements of the brain of a healthy volunteer reconstructed with the proposed method. Shown are reformatted views of the acquired volume in transversal, coronal and sagittal plane. *T*_1_ values given in milliseconds. Top left, fully sampled reference. From left to right and top to bottom increasing acceleration from 89 to 8 spokes per slice. Quantitative evaluation of ROIs is given in Table 3. The bottom half of the figure shows the corresponding error maps with a mask applied to the skull area.

**Figure 6:**
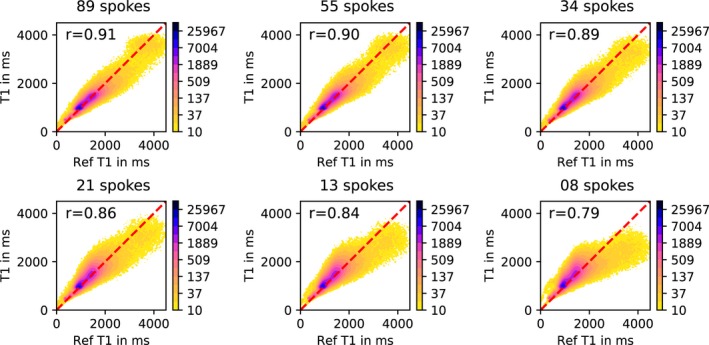
2D histogram contour plot of reference *T*_1_ values versus accelerated acquired and reconstructed *T*_1_ values for increasing acceleration using the VFA method. The color map encodes ares of mutual voxel values and is transformed using an exponential scaling. Red line indicates 45^∘^, corresponding to a perfect match, i.e. plotting the same data against each other. *r* indicates the Pearson correlation coefficient

The reconstructions of the full volume for the VFA approach are shown in Figure 5, displaying also the reformatted sagittal and coronal planes for a decreasing number of acquired spokes. For comparison, a *T*_1_ estimate from a fully‐sampled reconstruction is shown in the top left. For down to 21 spokes, the *T*_1_ maps show distinct edges with improved noise suppression in homogeneous areas throughout the whole volume. *T*_1_ mean and SD for specified ROIs are given in Table 3. Mean *T*_1_ values for accelerated reconstruction are contained in one SD of reference for down to 21 spokes. The accordance of the accelerated to the reference reconstructions is supported by error maps in the lower part of the figure. Only small errors are visible at the boundaries of CSF. The error, especially at tissue boundaries, increases with increasing acceleration, as visible in Figure 5. SSIM values of higher than 0.91 can be achieved for down to 21 acquired spokes. 2D histograms in Figure 6, showing dense clusters at *T*_1_ values for GM, WM and CSF, also support the quantitative accuracy compared to the reference for down to 21 spokes. Due to the proposed 3D regularization it was possible to decrease overall scan time from 21.5 s per slice to 1.8–1.1 s per slice for 34 respectively 21 spokes, depending on the accepted image degradation.

**Table 3:** Quantitative evaluation of 3D in vivo reconstructions for VFA and IRLL. All values are given as mean ± SD in ms. First part of the Table shows the quantitative evaluation for VFA brain measurements with ROIs specified in Figure 5. The second part lists the values for brain measurement with the IRLL method for ROIs given in Figure 7

	ROI 1	ROI 2	ROI 3	ROI 4	ROI 5	ROI 6
VFA						
Fully sampled	940 ± 75	935 ± 74	1482 ± 102	1433 ± 72	1323 ± 118	3826 ± 674
3D TGV						
89 Spokes	963 ± 35	944 ± 23	1479 ± 100	1440 ± 69	1356 ± 49	3843 ± 359
55 Spokes	969 ± 51	958 ± 33	1469 ± 116	1436 ± 110	1393 ± 73	3862 ± 432
34 Spokes	979 ± 63	951 ± 48	1412 ± 125	1433 ± 126	1415 ± 97	3572 ± 592
21 Spokes	982 ± 74	963 ± 66	1456 ± 129	1453 ± 157	1487 ± 136	3468 ± 694
13 Spokes	998 ± 85	967 ± 72	1470 ± 212	1512 ± 168	1447 ± 131	3647 ± 975
08 Spokes	1008 ± 79	989 ± 65	1658 ± 196	1582 ± 180	1509 ± 108	2866 ± 604
IRLL						
Fully sampled	793 ± 73	805 ± 77	1406 ± 172	1376 ± 120	1421 ± 169	2904 ± 271
8 s/slice TGV	786 ± 53	802 ± 61	1455 ± 201	1388 ± 190	1549 ± 239	3502 ± 1288
8 s/slice TV	786 ± 54	802 ± 61	1455 ± 201	1389 ± 190	1549 ± 239	3503 ± 1289
8 s/slice *L* ^1^‐wavelet	792 ± 151	806 ± 156	1465 ± 284	1419 ± 335	1623 ± 476	3548 ± 1339

**Figure 7:**
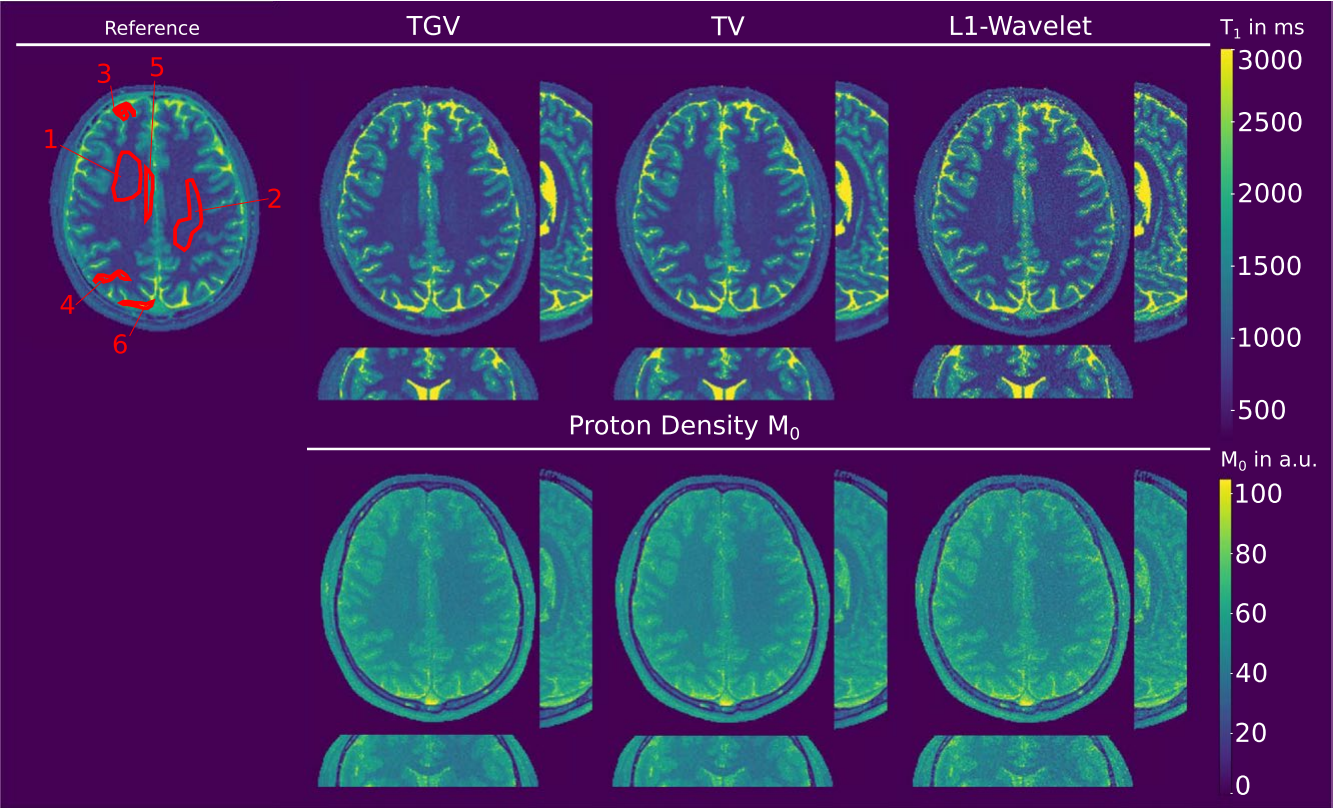
In vivo IRLL measurements of the brain of a healthy volunteer. Shown is a reformatted view of the acquired volume in transversal, coronal and sagittal plane. Top left, fully sampled Cartesian reference. In the upper row *T*_1_ reconstructions from data acquired in 8 s/slice. The pseudo proton density *M*_0_ is shown in the bottom row. Quantitative evaluation of ROIs for *T*_1_ is given in Table 3. *T*_1_ map values are given in milliseconds, *M*_0_ intensity values in arbitrary units (a.u.)

The reconstruction of the full volume for the IRLL approach is provided in Figure 7, showing the reformatted sagittal and coronal plane. *T*_1_ maps are in good agreement with the fully sampled Cartesian reference, generated with a model‐based framework in image space. *T*_1_ mean and SD for specified ROIs are given in Table 3 and lie within one SD from the fully sampled reconstruction, except for CSF. Exemplary 3D *M*_0_ maps are shown in the lower part of the figure. The proposed method was able to recover 3D *T*_1_ and *M*_0_ maps, acquired in 8 s per slice with 3D encoding and 1 mm^3^ isotropic resolution, compared to 110 s per slice for a fully sampled acquisition similar to the reference.

## 5 DISCUSSION

The proposed reconstruction framework for accelerated *T*_1_ quantification was successfully applied to highly undersampled, radially acquired VFA and IRLL data with 1 mm^3^ isotropic resolution. Validation of the algorithm was carried out with numerical, MRI phantom, and in vivo measurements. Substantial improvement in noise suppression compared to regularization was achieved with the proposed 3D TGV${}_{\text{Frob}}^{2}$ type regularization. Noise suppression is a well known behavior of TGV functionals in general^28^ while omitting “stair‐casing” artifacts and the proposed 3D Frobenius norm coupling takes full advantage of information from spatial and parametric space. In the present work, no stair‐casing was observable in the TV regularized reconstructions, yielding similar results compared to TGV based reconstruction. The structure of the reconstructed *T*_1_ maps, especially in the MRI phantoms, does support TV based regularization by showing large areas of flat *T*_1_ values separated by sharp edges, leading to a slight reduction of SD compared to TGV based reconstructions. The full potential of TGV can be exploited in areas with smoothly varying contrast which can be present in pathogenic tissue, e.g. brain tumors. The deterioration in performance of *L* ^1^‐wavelet regularization could be due to limitation to 2D wavelet computation since only a small amount of slices is available. Moreover, it was shown in^52^ that discrete difference based regularization marginally outperforms wavelets for cartilage *T*_1_ mapping, which agrees with our findings.

In vivo parameter maps from accelerated VFA data exhibit almost no detectable degradation for down to 34 spokes per slice (Figure 5). Further reduction of data results in increasing residual artifacts and a loss of quantitative accuracy, starting at 21 spokes. The influence on quantitative accuracy is clearly visible in the comparison of accelerated and fully sampled 2D histogram information (Figure 6), as well as in the error maps and ROI evaluations. Depending on the acceptable image quality, 21 to 34 spokes offer high acceleration with little to no loss in quantitative accuracy and image fidelity.

Reducing the amount of data can manifest in small deviations at tissue boundaries. Due to the reduced amount of data, structures at edges in the reconstructions are approximated by piecewise linear functions leading to a reduction of sharpness as explained in.^30^ A certain amount of SNR in the *k*‐space data is therefore mandatory in order to achieve reconstruction results with sufficient fidelity. This condition can be met by acquiring volumes, which is also of general diagnostic interest. Still, the used acquisition protocols lead to challenging SNR levels that were successfully tackled with the proposed reconstruction method.

For the proposed method it was observed that *T*_1_ values of small areas with low signal will be approximated by neighbouring values. This is observable for the border of the vials within the MR phantoms and in the skull area of the numerical simulations. However, corresponding *M*_0_ is close to background values (see Supporting Information Figure S4).

The reported mean and SD for *T*_1_ from VFA data, given in Table 1, are within one to two SD compared to the fully sampled reference. Tables 1 and 2 also support the visual intuition of increased SNR in reconstructions with the proposed algorithm, which can be seen by the reduced amount of SD in these reconstructions compared to regularization. Reported *T*_1_ values in selected ROIs of in vivo head data agree with values from literature.^2^ Slab profile effects can be seen at the top edge of the coronal view in Figure 5, leading to underestimated *T*_1_ values The piecewise linear approximation of structures from the TGV${}_{\text{Frob}}^{2}$ functional leads to systematic overestimation of low *T*_1_ values and an underestimation of high *T*_1_ values for in vivo acquisitions with thirteen and eight spokes, an effect also apparent in TV and wavelet based results. However, deviations are less than two SD, except for ROI 5, where a reconstruction artifact is present. The 3D isotropic nature of data acquisition is beneficial for *T*_1_ quantification in general, leading to reduced partial volume artifacts. The influence of 3D over 2D regularization is immanent as shown for VFA reconstructions in Figures 1, 2, 3, and Supporting Information Figure S3. The additional information exploited by regularizing on the whole 3D volume enables to reconstruct volumetric images from highly accelerated data, whereas 2D regularization results in higher proneness to noise, which manifests in discontinuities along the slice encoding direction (sagittal and coronal view). The 3D reconstruction approach stabilizes the ill‐posed optimization problem and removes discontinuities along the slice in the *T*_1_ maps for all investigated regularization strategies. Additionally we have shown that our method yields good results from prospectively accelerated data, a step often omitted. However, reconstruction from as few as eight spokes shows image degradation due to the limited amount of available data.

Opposed to the VFA method, the overall larger deviations for IRLL reconstruction compared to reference originate from the different acquisition techniques, i.e. 2D Cartesian vs 3D radial sampling. Care should be taken for short *T*_1_ values due to the binning of 13 spokes into one image frame, resulting in a temporal resolution of 65 ms, similar to 80 ms effective echo time of the 2D IRLL sequence. If accurate determination of short *T*_1_ values is important, temporal resolution can be reduced by binning fewer spokes into one frame without overall increase in scan time, a distinct advantage over Cartesian IRLL sequences. For IRLL, another interesting aspect can be exploited. In radial scans, every spoke contributes the same amount of information to the overall image contrast, a distinct difference to Cartesian scans. This contribution is included in the model by calculation of a mean according to the number of spokes‐per‐frame, reflecting the real data acquisition process more accurately, in contrast to the Cartesian case, where an apparent echo spacing has to be used.

Due to the multiplicative connection to the coil sensitivities, intensity inhomogeneities are corrected throughout optimization by *M*_0_ variations. This effect is visible in the frontal area of the transversal IRLL *M*_0_ map, whereas the *T*_1_ map is homogeneous in the corresponding area.

Compared to recent single shot model‐based reconstruction techniques for IRLL,^14^, ^15^ the proposed method offers high resolution 3D parameter maps with isotropic voxels. However, acquisition time is prolonged due to the necessity of sufficient relaxation after the readout even though the model accounts for shorter undisturbed relaxation.^32^, ^33^ Thus 8s/slice seemed to be the lower bound for accurate *T*_1_ estimations in 3D from IRLL data. The VFA method does not show such a limitation and enables *T*_1_ quantification in 1.8 s/slice equally to a almost 12‐fold reduction of scan time as compared to 6‐fold reduction as shown in recent work.^20^

Choosing the optimal regularization parameters is import for all iterative reconstruction techniques and usually specific for an imaging application. However, with the proposed normalization and scaling strategies, the regularization parameters, found after parameter training for the VFA and IRLL method, are in a similar range.

A common challenge for model‐based parameter quantification are relative long computation times^14^, ^15^ due to the repeated mapping from *k*‐space to parameter space. As a first step, the computationally expensive part of calculating the non‐uniform FFT is executed on the GPU, reducing reconstruction time by a factor of 20‐30 compared to pure python code. The reconstruction time for TGV${}_{\text{Frob}}^{2}$/TV/*L* ^1^‐wavelet based reconstruction depends heavily on the number of non‐uniform FFT computations, resulting in ∼9/6/9 min/slice for VFA data and ∼11/10/12 min/slice for IRLL data with the current implementation. Due to the same number of involved FFT computations, no increase in reconstruction time between 2D and 3D was observed. It is expected that a proper implementation of the framework using C/C++ and GPU programming will lead to further reduction of the reconstruction time.

## 6 CONCLUSIONS

With the proposed method it was possible to reconstruct 1 mm^3^ isotropic *T*_1_ maps from highly undersampled radially acquired data. Acquisition time could be reduced to 1.8–1.1 s/slice for VFA and 8 s/slice for IRLL data while preserving excellent quantitative accuracy. Reconstructions showed a gain in image fidelity compared to the fully sampled reference even for moderate to high acceleration. This was achieved by utilizing 3D TGV^2^ regularization combined with a spectral Frobenius norm to maximize the acceleration potential. We have further shown that the proposed solution strategy is applicable to different types of model‐based quantification problems.

## Supporting information


**FIGURE S1** Acquisition trajectories for the VFA and IRLL measurements. Top row shows an animated view, following the real acquisition trajectory, bottom row the full k‐space trajectory without any animation.
**FIGURE S2** Convergence rate over 13 GN steps for randomly chosen $T_{1}\in \left[200,\;5000\right]\;\text{ms}$ on a semi log scale. Values at *x* = 0 amount to the residual value after the first GN step. Data was normalized to yield an $L_{2}^{2}$‐norm of 1000.
**FIGURE S3** Error maps corresponding to the reconstructions in Figure 3.
**FIGURE S4** Exemplary *T*_1_ and *M*_0_ reconstruction for the VFA phantom acquired with 21 radial spokes. Colormap is scaled between minimal and maximal occurring *M*_0_ values. Areas with little to no signal are showing *M*_0_ values close to the background and a simple threshold could be used to mask out these areas in the corresponding *T*_1_ map. Due to *M*_0_ being influenced by technical and physiological factors neglected in the signal equation such as *T*_2_* and coil sensitivity variations, inhomoegneites in *M*_0_ can be introduced as seen in this exemplary reconstruction.
**TEXT S1** Computational complexity analysis for one iteration of the described PD algorithm within a GN step.

## APPENDIX 1 Numerical solution

As described in Section “Numerical Solution” it is our goal to solve the following optimization problem within each GN iteration

(12) $${\hat{u}}_{k}\;=\;\underset{u,v}{\text{arg min}}\;\frac{1}{2}\|{\text{DA}}_{k}u-d_{k}{\|}_{2}^{2}+\lambda \left(\alpha_{1}{\left\|\nabla u-v\right\|}_{1,2,2}+\alpha_{0}{\left\|Ev\right\|}_{1,2,2}\right)+\frac{1}{2\gamma }{\left\|u-u_{k}\right\|}_{2}^{2},$$

where $\nabla :U^{N_{u}}\mapsto U^{3\;\times \;N_{u}}$ and $E:U^{3\;\times \;N_{u}}\mapsto U^{6\;\times \;N_{u}}$ are defined as

$$\nabla u\;=\;{\left(\delta_{\text{x}+}u^{i},\delta_{\text{y}+}u^{i},\delta_{\text{z}+}u^{i}\right)}_{i\;=\;1}^{N_{u}}$$

and

$$Ev\;=\;{\left(\delta_{\text{x}-}v^{1,i},\delta_{\text{y}-}v^{2,i},\delta_{\text{z}-}v^{3,i},\frac{\delta_{\text{y}-}v^{1,i}+\delta_{\text{x}-}v^{2,i}}{2},\frac{\delta_{\text{t}-}v^{1,i}+\delta_{\text{x}-}v^{3,i}}{2},\frac{\delta_{\text{z}-}v^{2,i}+\delta_{\text{y}-}v^{3,i}}{2}\right)}_{i\;=\;1}^{N_{u}}.$$

The operators *δ*_x+_, *δ*_y+_, *δ*_z+_ and *δ*_x−_, *δ*_y−_, *δ*_z−_ define symmetrically extended forward and backward finite difference operators, respectively, with respect to the x, y and z coordinate. To compute the update steps of the PD algorithm as

(13) $$\begin{array}{cc} y^{n+1} & \;=\;{\left(I+\sigma \partial F*\right)}^{-1}\left(y^{n}+\sigma K{\bar{x}}^{n}\right)\\ x^{n+1} & \;=\;{\left(I+\tau \partial G\right)}^{-1}\left(x^{n}-\tau K^{H}y^{n+1}\right)\\ {\bar{x}}^{n+1} & \;=\;x^{n+1}+\theta \left(x^{n+1}-x^{n}\right) \end{array}$$

the following operations need to be defined.

The adjoint operations of *K*,* K* ^*H*^ are

(14) $$K^{H}\;=\;\left(\begin{array}{ccc} {\text{DA}}_{k}^{H} & -{\text{div}}^{1} & 0\\ 0 & -Id & -{\text{div}}^{2} \end{array}\right),$$

where the divergence operators div^1^ and div^2^ are the negative adjoints of ∇ and E, respectively. The adjoint of DA_*k*_ reads as

$${\text{DA}}_{k}^{H}:\xi \;=\;{\left(\xi_{p}\right)}_{p\;=\;1}^{N_{p}}\to {\left(\sum_{p\;=\;1}^{N_{p}}{\left.\bar{\frac{\partial S_{p}\left(u\right)}{\partial u_{i}}}\right|}_{u\;=\;u_{k}}\sum_{c\;=\;1}^{N_{c}}\bar{b_{c}}F^{-1}\left[\xi_{p}\right]\right)}_{i\;=\;1}^{N_{u}}.$$

The operators *P* corresponding to the proximal mapping of *F**, i.e. the convex conjugate of *F*, and *G* in the algorithm are given by

$$P_{\alpha_{0},\alpha 1}{\left(\xi \right)}_{j,k,l}\;=\;\frac{\xi_{j,k,l}}{\max\left(1,\frac{|\xi_{j,k,l}|}{\alpha_{0}\lambda ,\alpha 1\lambda }\right)},\;P_{L^{2}}\left(\xi \right)\;=\;\frac{\xi -\sigma d_{k}}{1+\sigma },\;\text{and}\;P_{G}\left(\xi \right)\;=\;\frac{\frac{\tau }{\gamma }u_{k}+\xi }{1+\frac{\tau }{\gamma }}$$
