# Supplementary material for: Rapid T1 quantification from high resolution 3D data with model‐based reconstruction
Source: Magn Reson Med. 2018 Oct 22;81(3):2072–89. doi: 10.1002/mrm.27502 (PMC6588000; doi:10.1002/mrm.27502)
Supplement: Supplementary file 1 — FIGURE S1 Acquisition trajectories for the VFA and IRLL measurements. Top row shows an animated view, following the real acquisition trajectory, bottom row the full k‐space trajectory without any animation. FIGURE S2 Convergence rate over 13 GN steps for randomly chosen T1∈[200,5000]ms on a semi log scale. Values at x = 0 amount to the residual value after the first GN step. Data was normalized to yield an L22‐norm of 1000. FIGURE S3 Error maps corresponding to the reconstructions in Figure 3. FIGURE S4 Exemplary T 1 and M 0 reconstruction for the VFA phantom acquired with 21 radial spokes. Colormap is scaled between minimal and maximal occurring M 0 values. Areas with little to no signal are showing M 0 values close to the background and a simple threshold could be used to mask out these areas in the corresponding T 1 map. Due to M 0 being influenced by technical and physiological factors neglected in the signal equation such as T 2* and coil sensitivity variations, inhomoegneites in M 0 can be introduced as seen in this exemplary reconstruction. TEXT S1 Computational complexity analysis for one iteration of the described PD algorithm within a GN step. [file MRM-81-2072-s001.pdf]

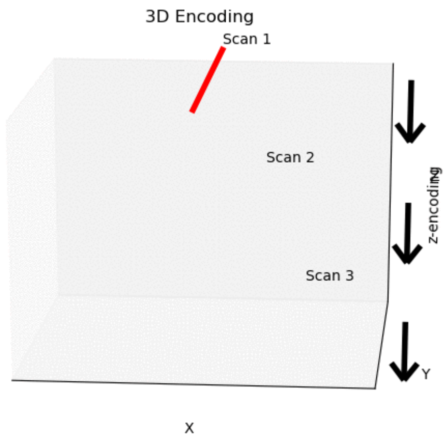

(a) VFA

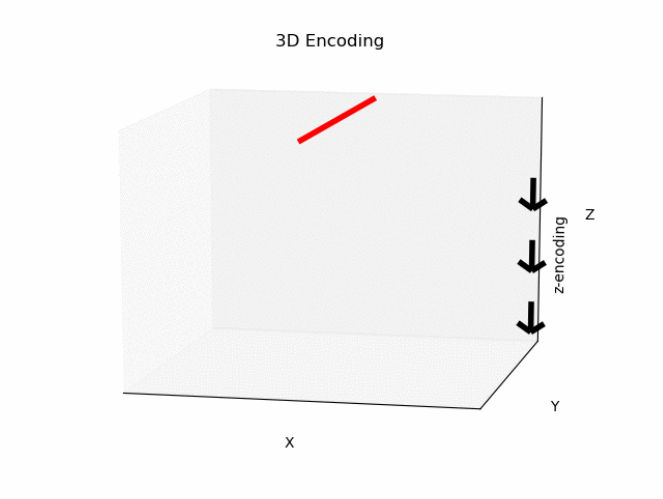

(b) IRLL

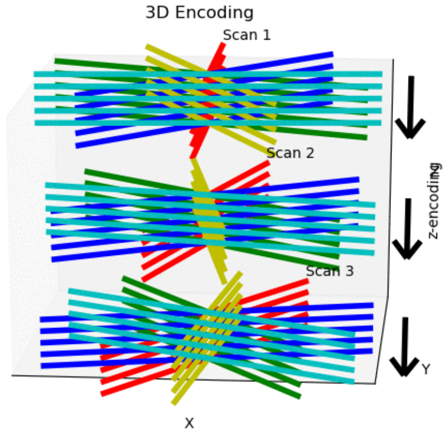

(c) VFA

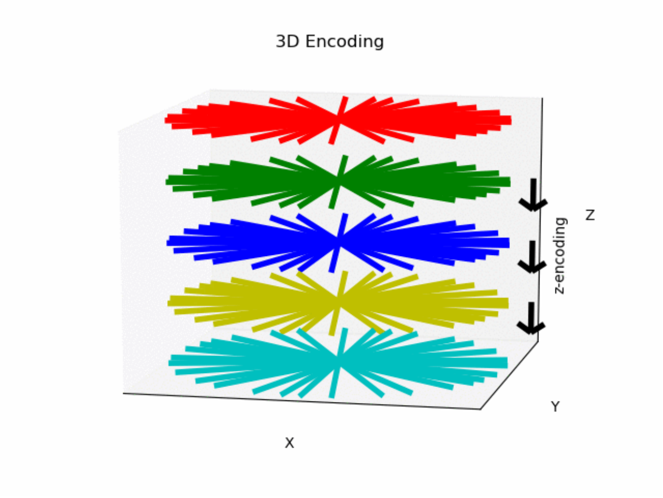

(d) IRLL

Supporting Information Figure S1: Acquisition trajectories for the VFA and IRLL measurements. Top row shows an animated view, following the real acquisition trajectory, bottom row the full k-space trajectory without any animation.

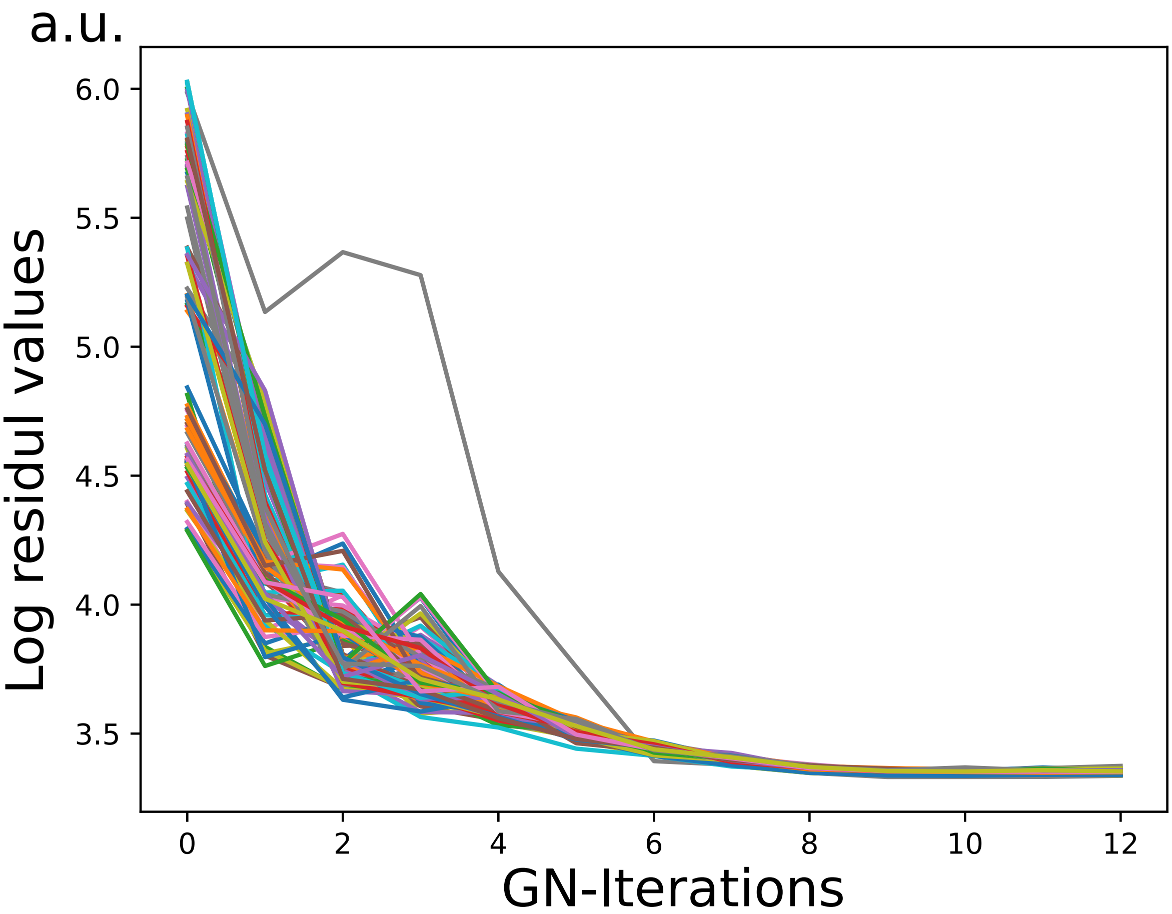

Supporting Information Figure S2: Convergence rate over 13 GN steps for randomly chosen $T_1 \in [200, 5000]ms$ on a semi log scale. Values at $x = 0$ amount to the residual value after the first GN step. Data was normalized to yield an L_2^2-norm of 1000.

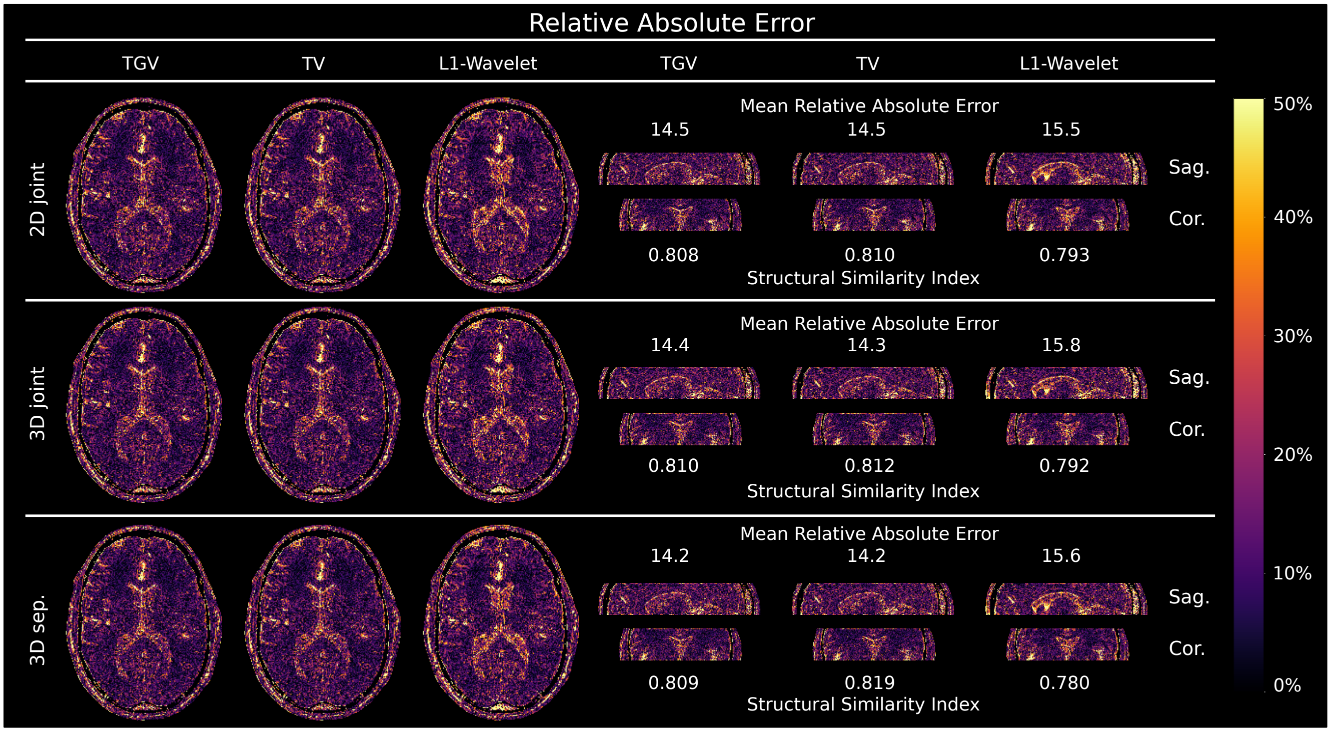

Supporting Information Figure S3: Error maps corresponding to the reconstructions in Figure 3.

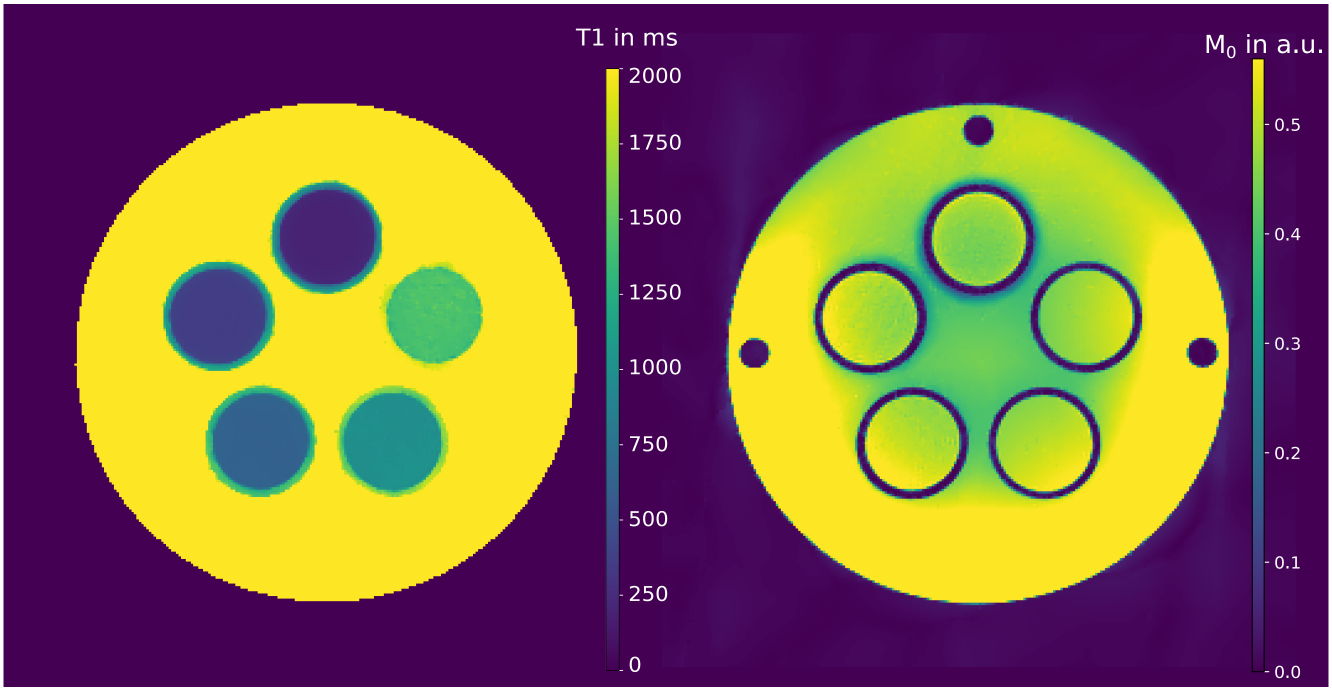

Supporting Information Figure S4: Exemplary T_1 and M_0 reconstruction for the VFA phantom acquired with 21 radial spokes. Colormap is scaled between minimal and maximal occurring M_0 values. Areas with little to no signal are showing M_0 values close to the background and a simple threshold could be used to mask out these areas in the corresponding T_1 map. Due to M_0 being influenced by technical and physiological factors neglected in the signal equation such as T_2^* and coil sensitivity variations, inhomoegneites in M_0 can be introduced as seen in this exemplary reconstruction.

COMPUTATIONAL COMPLEXITY

Dimensions:

$$X = N_x$$
$$M = N_z \times N_c \times N_p$$
$$U = N_z \times N_u$$
$$N = N_d$$

Operations:

$$\mathcal{O}(NFFT) = \mathcal{O}\left(M[N \log N]\right) \qquad \mathcal{O}(S(u)) = \mathcal{O}(UX^2)$$
$$\mathcal{O}(\nabla S(u)) = \mathcal{O}(UX^2) \qquad \mathcal{O}(DAu) = \mathcal{O}\left(M[N \log N] + UX^2\right)$$
$$\mathcal{O}(DA^T u) = \mathcal{O}\left(M[N \log N] + UX^2\right) \qquad \mathcal{O}(\nabla u) = \mathcal{O}(UX^2)$$
$$\mathcal{O}(\mathcal{E}u) = \mathcal{O}(UX^2) \qquad \mathcal{O}(\|\cdot\|_{parameter\,space}) = \mathcal{O}(UX^2)$$
$$\mathcal{O}(\|\cdot\|_{k-space}) = \mathcal{O}(NM)$$

Primal and Dual updates:

$$\mathcal{O}((Id + \tau\partial G)^{-1}(x - \tau K y)) = \mathcal{O}\left(M[N \log N] + UX^2\right)$$
$$\mathcal{O}((Id + \sigma\partial F^*)^{-1}(x + \sigma K^T x)) = \mathcal{O}\left(M[N \log N] + UX^2\right)$$

Overall Complexity of the algorithm:

$$\mathcal{O}\left(M[N \log N] + UX^2\right)$$

Supporting Information Text S1: Computational complexity analysis for one iteration of the described PD algorithm within a GN step.
